# Functionalization of Commercial Electrospun Veils with Zinc Oxide Nanostructures

**DOI:** 10.3390/nano11020418

**Published:** 2021-02-06

**Authors:** Irene Bavasso, Francesca Sbardella, Maria Paola Bracciale, Matteo Lilli, Jacopo Tirillò, Luca Di Palma, Anna Candida Felici, Fabrizio Sarasini

**Affiliations:** 1 Department of Chemical Engineering Materials Environment, Sapienza-Università di Roma & UdR INSTM, Via Eudossiana 18, 00184 Roma, Italy; francesca.sbardella@uniroma1.it (F.S.); mariapaola.bracciale@uniroma1.it (M.P.B.); matteo.lilli@uniroma1.it (M.L.); jacopo.tirillo@uniroma1.it (J.T.); fabrizio.sarasini@uniroma1.it (F.S.); 2 Department of Basic and Applied Sciences for Engineering, Sapienza-Università di Roma, Via Scarpa 16, 00161 Roma, Italy; annac.felici@uniroma1.it

**Keywords:** ZnO, electrospun polymer veil, nylon, wurtzite nanorods, photocatalytic activity

## Abstract

The present research is focused on the synthesis of hexagonal ZnO wurtzite nanorods for the decoration of commercially available electrospun nylon nanofibers. The growth of ZnO was performed by a hydrothermal technique and for the first time on commercial electrospun veils. The growth step was optimized by adopting a procedure with the refresh of growing solution each hour of treatment (Method 1) and with the maintenance of a specific growth solution volume for the entire duration of the treatment (Method 2). The overall treatment time and volume of solution were also optimized by analyzing the morphology of ZnO nanostructures, the coverage degree, the thermal and mechanical stability of the obtained decorated electrospun nanofibers. In the optimal synthesis conditions (Method 2), hexagonal ZnO nanorods with a diameter and length of 53.5 nm ± 5.7 nm and 375.4 nm ± 37.8 nm, respectively, were obtained with a homogeneous and complete coverage of the veils. This easily scalable procedure did not damage the veils that could be potentially used as toughening elements in composites to prevent delamination onset and propagation. The presence of photoreactive species makes these materials ideal also as environmentally friendly photocatalysts for wastewater treatment. In this regard, photocatalytic tests were performed using methylene blue (MB) as model compound. Under UV light irradiation, the degradation of MB followed a first kinetic order data fitting and after 3 h of treatment a MB degradation of 91.0% ± 5.1% was achieved. The reusability of decorated veils was evaluated and a decrease in photocatalysis efficiency was detected after the third cycle of use.

## 1. Introduction

Nowadays, the adoption of fiber reinforced polymers (FRPs) represents a valid alternative to traditional homogeneous materials thanks to their high specific mechanical properties [1]. The main limitation of such reinforced materials is their poor delamination strength [2], inherited from their anisotropic and heterogeneous nature. This results in poor impact damage resistance and tolerance, thus affecting the long-term reliability of polymer composites especially when brittle thermosetting polymer is adopted as matrix [3]. Among the many techniques used to face this issue [4,5,6,7,8], the current trend is to add a thermoplastic toughening phase (films, nanoparticle, and non-woven veils) in the thermosetting polymeric matrix [9,10]. Some of these approaches have shortcomings. For instance, toughening particles often result in a non-homogeneous dispersion that affects the performance of the composites and limits the application of laminates [11]. Interleaving veils in polymer matrix, especially with fibers at the nanoscale, is considered as one of the most promising toughening methods thanks to the high surface area to volume ratio, flexibility in surface functionalities, small-size pores, and excellent mechanical performance [3] compared to microfibers of the same material that can result in an increase in weight and thickness of the final laminate [11]. The maintenance of nano-dimension in large-scale production can be ensured by the adoption of high electrostatic fields capable of producing ultrafine fibers from the polymer solution [12]. The first inclusion of toughening materials fabricated by electrospinning into middle plies of the composites was reported in 1999, when electrospun nanofibers of poly-benzimidazole were successfully used as nonwoven fabric reinforcements in epoxy and rubber matrices [13]. By considering the hierarchical nature of the resulting laminated composites, their properties are intrinsically dependent on the interaction between the nanofibrous veils and the thermosetting resin that, for example, sometimes did not contribute to a noticeable improvement of the critical strain energy release rate (*G_Ic_*) during Mode I loading condition [14]. This effect can be attributed also to nanofiber physical [15] and chemical properties [11]. In an attempt to solve the problem of interfacial adhesion between the electrospun fibers and matrix, surface modification of the fibers with the integration of inorganic nanostructures could be a solution for the production of high-performance materials. The addition of inorganic nanoparticles improves the mechanical and thermal performances of the organic substrate and, in addition, provides the polymer matrix with the properties typical of the nanoparticles (optical, electrical, antimicrobial, antifouling, and catalytic) [16]. Among these inorganic nanostructures, semiconductor ZnO is an attractive low-cost and non-toxic material. It has excellent properties such as wide band gap (*E_g_* = 3.37 eV) [17], high thermal conductivity and an inherent ability to exhibit different shapes and morphologies depending on the synthesis parameters [18]. Thermal evaporation [19], metal-organic chemical [20] or thermo-chemical [21] vapor deposition have been proposed for ZnO nanostructures fabrication, but all of these techniques are complex and involve high process temperature (>350 °C) [22]. The whiskerization approach leads to an increase in the toughness of the composites while a decrease of strength is observed, because of the high temperature involved during the synthesis of nanostructures [23]. To reduce this issue, Lin and co-workers have proposed a low-temperature (<90 °C) hydrothermal procedure and an increase of 113% in interfacial strength of carbon fiber modified by whiskerization technique was observed without damaging the fibers [23]. The growth of ZnO nanostructures on electrospun nanofibers has been proposed by several authors under different conditions [24]. For example, Athauda et al. demonstrated the feasibility of ZnO nanowires on electrospun cellulose acetate/polyvinyl acetate/polyethylene glycol mats through hydrothermal technique [17]. Preda et al. prepared poly(methyl methacrylate) electrospun fiber mats decorated with ZnO prisms through electroless deposition [25]. A considerable interest is focused on polyamides as synthetic matrix with good strength and chemical resilience [26], which can be decorated with ZnO structures such as nanowires [27], rods [28], and nanorods [29] when the polymer is used in fiber form [27,29] or as electrospun fiber [28]. In [28] the authors reported the synthesis of mop-brush-shaped ZnO rods over nylon-6 electrospun nanofibers by incorporating ZnO nano-seeds directly in the electrospun nylon-6 fibers. Although the hydrothermal procedure has already been applied to electrospun materials, as previously discussed [17,18,19,20,21,22,23,24,25,26,27,28], the coverage was not particularly homogeneous, and the authors did not investigate the thermal and mechanical properties of the resulting mats. Athauda et al. [30] have for the first time successfully demonstrated the possibility of growing hexagonal wurtzite ZnO on self-made electrospun nylon veil by the hydrothermal method as low cost and easily extendable to large-scale production technique, but no description about the polymer modification during the chemical and thermal treatment is reported. Our purpose was to investigate whether such technique could be adopted also for commercial electrospun veils whose chemical/physical characteristics are not fully specified. This is the first study on the growth of ZnO nanostructures on a commercial material. In particular, with the aim of defining a simple and scalable procedure, in this paper the optimization of ZnO morphology by working on several process parameters of the hydrothermal procedure, such as the overall treatment time and the volume of growth solution, is presented. Such optimization takes into account not only the homogeneous distribution over the electrospun nanofibers, but also the evaluation of mechanical performance and thermal stability of the resulting hierarchical mats. In this study, the effects induced by the alkaline and alcoholic solutions used during the ZnO synthesis on the mechanical, thermal, and morphological properties of the resulting decorated non-woven mats have been investigated. Moreover, the optimized hydrothermal growth strategy reported in this work allowed to achieve decorated nanofibers with higher amounts by weight of ZnO compared to other studies performed on nylon microfibers [27,28,29]. These hierarchically nanostructured mats could be potentially exploited as enhancers of through-the-thickness properties of the resulting composites under low-velocity impact conditions. Since the ZnO nanorods are stiff structures, they are expected to protrude into the matrix material and improve load transfer between the fiber and matrix (enhancement in the interlaminar resistance).

The presence of photocatalytic species, such as the ZnO nanostructures, makes the decorated veils also attractive for wastewater remediation. Thanks to the presence of nanostructures attached to the veil (support), the adoption of a separation step for the recovery of the nanoparticles at the end of the treatment is prevented [31]. To investigate the environmental application of such materials, photocatalytic tests were performed to evaluate the degradation of methylene blue (MB) used as a model compound under UV light irradiation.

## 2. Materials and Methods

### 2.1. Materials

Zinc acetate dihydrate (Zn(CH_3_COO)_2_·2H_2_O, ACS reagent, ≥98%), sodium hydroxide (NaOH, ACS reagent, ≥97.0%, pellets), zinc nitrate hexahydrate (Zn(NO_3_)_2_·6H_2_O, purum p.a., crystallized, ≥99.0%), hexamethylenetetramine (C_6_H_12_N_4_, ACS reagent, ≥99.0%), ethanol absolute (C_2_H_5_OH), and methylene blue (C_16_H_18_CIN_3_S) were purchased from Sigma-Aldrich (St. Louis, MO, USA) and used without any further purification. The ZnO growth was conducted on commercially available electrospun veils provided by RevolutionFibres (Xantu.Layr^®^, Auckland, New Zealand) with an areal density of 4.5 g/m^2^ and a thickness around 15 μm [32].

### 2.2. Hydrothermal Growth of ZnO Nanostructures on Electrospun Veils

The hydrothermal growth of ZnO nanostructures was performed according to the method proposed by Galan et al. [33]. Briefly, this synthesis involves two stages, namely seeding and growth steps. For the seeding step, two ethanol-based solutions of Zn(CH_3_COO)_2_·2H_2_O (0.0125 M) in 80 mL and NaOH (0.002 M) in 100 mL were used. Both solutions were maintained under vigorous stirring at 50 °C for 5 min. The solutions were cooled till reaching the room temperature and 40 mL of both zinc and sodium hydroxide solutions were made up to 360 mL and 140 mL, respectively, with ethanol. Then the solutions were heated to 65 °C and mixed together for 30 min by keeping constant the temperature. Before the seeding, the electrospun veils were soaked in ethanol for 10 min and then dried at 100 °C as a pre-treatment. Then, they were dipped into the seed solution for 10 min and annealed at 150 °C for 10 min. This procedure allows to immobilize ZnO seed onto electrospun nanofibers to provide growth using homoepitaxy [34,35] followed by the hydrothermal process.

For the growth step, a solution of ultrapure water with equimolar concentration of Zn(NO_3_)_2_·6H_2_O and C_6_H_12_N_4_ (0.0249 M) was prepared and heated at 90 °C. This solution was used to dip the electrospun veils without changing the temperature for a time in a range from 1 to 5 h. The mechanism involved in the growth process was studied by several authors [22,23,24,25,26,27,28,29,30,31,32,33,34,35,36] and reported in Equations (1)–(5). The high temperature supports the decomposition of the two precursors and provides the dissolution of the colloidal Zn(OH)_2_ that contributes as nuclei for the growth of ZnO [22].

(1) $$\mathrm{Zn}({\mathrm{NO}}_{3}{)}_{2}\;+\;6H_{2}O\;\to \;{\mathrm{Zn}}^{2+}\;+\;2{{\mathrm{NO}}_{3}}^{-},$$

(2) $$C_{6}H_{12}N_{4}\;+\;6H_{2}O\;\to \;6\mathrm{HCHO}\;+\;4{\mathrm{NH}}_{3},$$

(3) $${\mathrm{NH}}_{3}\;+\;H_{2}O\;\to \;{{\mathrm{NH}}_{4}}^{+}\;+\;{\mathrm{OH}}^{-},$$

(4) $${\mathrm{Zn}}^{2+}\;+\;2{\mathrm{OH}}^{-}\;\to \;\mathrm{Zn}(\mathrm{OH}{)}_{2},$$

(5) $$\mathrm{Zn}(\mathrm{OH}{)}_{2}\;\to \;\mathrm{ZnO}\;+\;H_{2}O$$

In this work the growing step was executed following two procedures: (i) Method 1 was based on refreshing every hour the growth solution preheated at 90 °C; (ii) in Method 2 definite volumes (75 mL and 250 mL) of growth solutions were adopted without changing the growth solution. At the end of the growth step the veils were washed with ultra-pure water and dried at 60 °C. Tests at different times of the growth step as 1, 3, and 5 h were conducted following the Method 1. In the case of Method 2, the veils were decorated by treating them at selected times, i.e., 3 and 5 h. The synthesis reproducibility has been validated against at least three replicates performed at different times.

### 2.3. Characterization of Neat and Decorated Electrospun Veils

The occurrence of ZnO nanostructures growth and their morphology were investigated by scanning electron microscopy (SEM, MIRA3 by Tescan, Brno, Czech Republic) equipped with energy dispersive spectroscopy (EDAX) and operated at 5.0 kV. Before the analysis, the specimens were sputter coated with gold. Several micrographs were examined to measure the diameter and the length of ZnO nanostructures by using the ImageJ software. All measures, recorded manually, have been elaborated in order to define an average value and a standard deviation for at least 50 nanorods for each condition. To investigate the crystalline structure of ZnO nanostructures, X-ray diffraction (XRD, Philips X’Pert Pro, PANalytical B.V., Almelo, The Netherlands) analysis was performed in a continuous scan mode in the 2θ range from 10° to 80° with a step size of 0.02° and a time per step of 3 s. The monochromatic radiation adopted was Cu Kα (40 Kv–40 mA).

The effect of ZnO synthesis procedure on the thermal stability and mechanical properties of electrospun veils was assessed by thermogravimetric analysis (TGA) and tensile tests, respectively. Analysis of the mass loss of untreated and treated veils with temperature was carried out using a thermogravimetric analyzer (SetSys Evolution, Setaram Instrumentation, Caluire, France) at a heating rate of 10 °C/min to a maximum temperature of 800 °C in a nitrogen atmosphere.

Tensile properties of neat and ZnO-decorated electrospun veils were determined in accordance with UNI EN ISO 527-2 using type 1BA samples (l_0_ = 30 mm). Tests were carried out in displacement control with a crosshead speed of 10 mm/min on a Zwick/Roell Z010 (Zwick/Roell GmbH, Ulm, Germany).

Brunauer–Emmett–Teller (BET) analyses were performed by N_2_ adsorption isotherms acquired at −196 °C using a Micromeritics Triflex analyzer (Micromeritics Instrument Corp. Norcross (Atlanta), GA, USA) in the p/p_0_ range from 0.01 to 0.99. Isotherm analyses were carried out using the 3Flex Version 4.05 software. Samples were previously outgassed at 100 °C for 3 h. The BET equation was used to determine the specific surface area.

Diffuse reflectance UV–Vis spectra (UV-vis-Diffuse Reflectance Spectroscopy) to determine the band gap of the ZnO photocatalyst were carried out using a spectrophotometer (AvaSpec-2048, Avantes, Apeldoorn, The Netherlands) equipped with a halogen lamp with a tungsten filament (HL-2000 FHSA, Avantes, Apeldoorn, The Netherlands) as light source. The reflectance measurements were collected with a spectral resolution of 0.8 nm between 300 and 1100 nm with an integration time of 40 ms and 100 scans. A Spectralon standard (Labshere SRS-99-010, 99% reflectance, North Sutton, NH, USA) was taken as reference for the reflectance spectra.

### 2.4. Photocatalytic Degradation of Methylene Blue

The application of the synthesized materials in water remediation was evaluated by performing photocatalytic experiments in a glass reactor filled by 18 mg/L of methylene blue (MB) solution. The veils (2.5 cm × 2.5 cm and 5.7 mg) decorated with ZnO nanostructures were placed into a reactor whose top was maintained open and irradiated by an UV source (365 nm and irradiance_max_ = 20 W/m^2^). The distance between the reactor and the UV source was 13 cm. The MB concentration was monitored as a function of time by measuring its highest absorption peak (666 nm) with a PG Instruments T80+ UV/Vis spectrophotometer (using a glass cell of 1 cm path length, Leicestershire, UK). To evaluate the mineralization of MB, Total Organic Carbon (TOC) analyses were conducted at the beginning and at the end of each test by using a Shimadzu TOC-L CSH/CSN analyzer (Milan, Italy). The MB degradation and mineralization (%) were calculated according to the following equation:

*A*(%) = (*A*_0_ − *A_t_*)/*A*_0_ × 100, (6)

where *A*_0_ is the absorbance or the TOC at initial time, while *A_t_* at a generic time *t*.

To estimate the performance of decorated veils after several cycles of use, different photocatalytic tests were performed by using the same veil. The efficiency of photocatalytic species was evaluated by comparing the MB degradation with those obtained using commercial nano TiO_2_ (P25, nanopowder, 21 nm, provided by Sigma Aldrich, St. Louis, MO, USA) at a content equal to the ZnO nanorods found in decorated veils.

## 3. Results and Discussion

### 3.1. Morphological, Thermal, and Mechanical Properties

#### 3.1.1. Method 1

**Figure 1:**
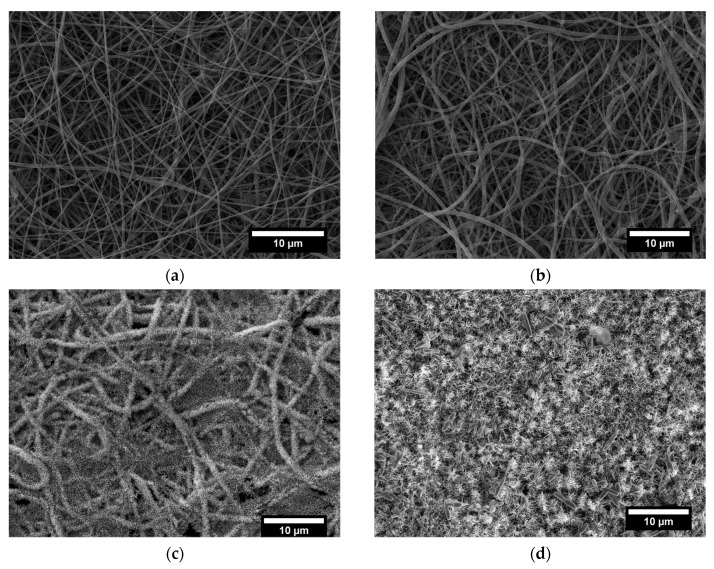
SEM micrographs of (**a**) neat commercial electrospun veil and veils decorated with ZnO nanostructures with Method 1 procedure at (**b**) 1 h, (**c**) 3 h, and (**d**) 5 h growth treatment time.

The first part of this study dealt with the identification of the best operating conditions for the decoration of commercial electrospun veils with ZnO nanostructures. During the growth step performed following the Method 1 procedure, the solution was refreshed after each hour and the number of cycles (overall growth time) was the main parameter to be defined. In Figure 1 the SEM micrographs of neat commercial electrospun veil and the veils after ZnO growth step in Method 1 are reported.

The as-received veil (Figure 1a) was formed by randomly oriented nanofibers with an average diameter of 196.6 nm ± 77.5 nm and a smooth surface. The seed treatment of nylon nanofibers provides the creation of nucleation sites for the subsequent growth of single crystal ZnO nanorods and the absence of the seeding step could result in uncontrolled growth of nanostructures with high aspect ratio not complying with nanorods category [37]. For this reason, in the present work only the seed assisted procedure was adopted.

**Figure 2:**
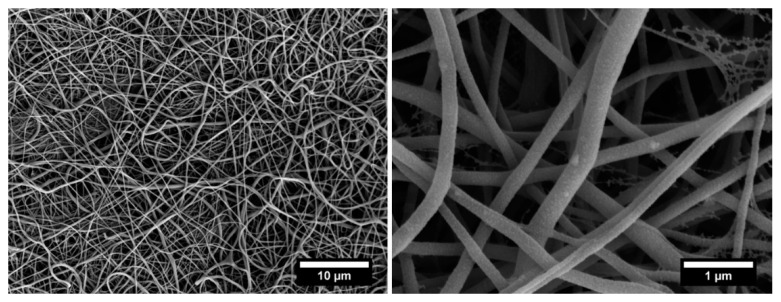
SEM micrographs of ZnO seed deposited nylon nanofibers at different magnifications.

Figure 2 presents the SEM micrographs of ZnO seed deposited on nylon veils, where it can be observed that ZnO nanoparticles are uniformly distributed over the entire nanofiber surfaces.

The presence of Zn-based nanoparticles on nylon nanofibers was confirmed by energy dispersive X-ray (EDX) spectroscopy (Figure S1), which shows the appearance of zinc (Zn) atoms apart from the basic chemical elements of nylon polymer as carbon (C), oxygen (O), and nitrogen (N) atoms (with the exception of (Au) atoms coming from the gold coating layer).

**Figure 3:**
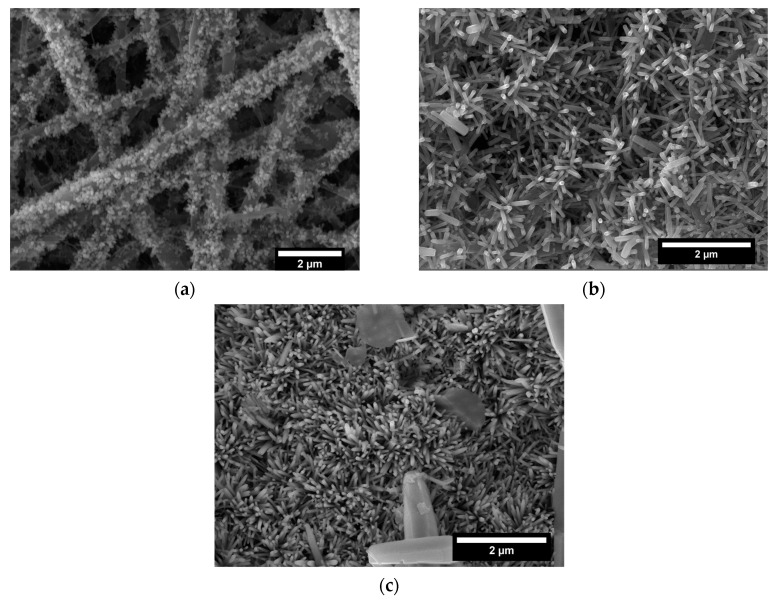
Magnified SEM micrographs of veils decorated with ZnO nanostructures with Method 1 procedure at (**a**) 1 h, (**b**) 3 h and (**c**) 5 h growth treatment time.

The SEM micrographs after the ZnO growth (Figure 1c,d) revealed the presence of nanostructures radially oriented and evenly distributed on the nanofibers surface. In particular, already in one hour of treatment it is possible to obtain a good coverage of the nanofibers with ZnO nanostructures (Figure 3a). However, one hour was not enough to ensure the development of vertically grown nanorods, and only a measurement of the diameter was possible (82.0 nm ± 18.5 nm, Figure S2). After 3 h of treatment, a diameter of 70.5 nm ± 11.5 nm and height of 362.4 nm ± 104.6 nm were measured while, after 5 h, a quite limited increase of diameter and height of ZnO nanostructures of 86.3 nm ± 21.3 nm and 364.7 nm ± 125.7 nm, respectively, was observed on average (Figure S2). Moreover, an abnormal growth of zinc oxide structures was found after the fifth cycle of treatment with the development of coarse deposits on the surface of the electrospun veil (white arrows in Figure 3c). This effect can be ascribed to the reactions occurring in the formation of ZnO nanostructures. As reported before, the ZnO precipitation follows the mechanism with Zn(OH)_2_ as intermediate and the distribution of all complexes produced during the growth process is closely dependent on the concentration of the Zn^2+^, pH and temperature [38]. During the reaction, the refresh of the growth solution guaranteed a high concentration of the zinc ions and the presence of the hexamethylenetetramine acted as buffer to prevent any pH variation [39]. When the high temperature (90 °C) is maintained, the hydrolysis of Zn(OH)_2_ occurred and the consequent growth of the ZnO nanorods in ensured. However, during the Method 1 procedure, the refresh of the growth solution is operated in manual mode, and it may lead, although for a short time, to a decrease in the operative temperature. At low temperature, the thermodynamic stability of Zn(OH)_2_ [40] and the slowdown of the hydrolysis reaction [41] involve the precipitation of both hydroxide and coarse ZnO.

**Figure 4:**
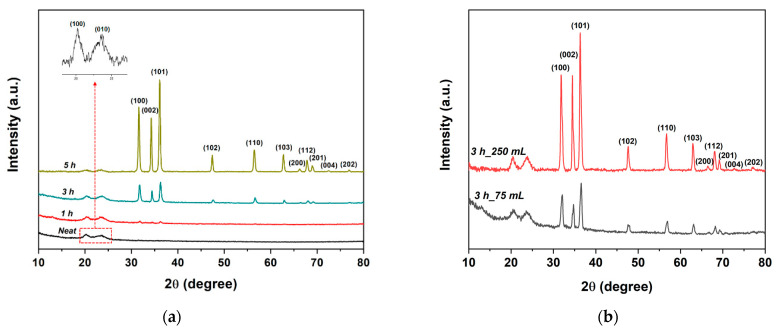
XRD patterns of (**a**) neat and ZnO-decorated electrospun veils at different growth treatment times in Method 1 and (**b**) ZnO-decorated electrospun veils at different growth treatment volumes in Method 2.

The crystalline structure of the samples was investigated by XRD analysis, as shown in Figure 4a. In the recorded diffraction pattern of neat veil, the two peaks at 2θ of 20.23° (100) and 23.83° (010) represent a distinctive feature of the α-phase of triclinic nylon 6,6 [42,43], with no evidence of the γ-phase [44]. After the growth step, eleven new reflection peaks appeared, which can be indexed as the hexagonal wurtzite structure of ZnO [45] (JCPDS 79-0207). The intensity of such peaks increased with increasing growth time, thus suggesting that ZnO nanorods are well crystallized and that the growth step at mild temperatures did not compromise the quality of the ZnO crystals observed on the surface of the electrospun veils, as confirmed by other works [17]. In addition, no notable changes in the crystalline structure of pristine nylon were observed. The formation of impurity phases was excluded since no other diffraction peaks were detected.

**Figure 5:**
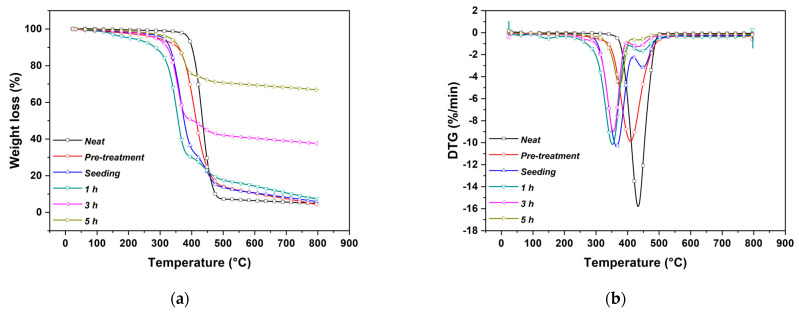
(**a**) Thermograms and (**b**) first derivative weight loss of neat electrospun veil and ZnO-decorated veils at different growth treatment times in Method 1.

The amount of ZnO crystals on the surface of the electrospun veils increased with time, as suggested by TGA analysis reported in Figure 5.

Specimens showed a similar first limited decomposition (Figure 5a) in the range 50–100 °C ascribed to the evaporation of surface-bound water (natural moisture). The electrospun veils thermal degradation occurred in a single-stage decomposition (370.91–508.59 °C) due to the tendency of adipic acid residue segments in nylon 6,6 to cyclize [46], and to the crosslinking reaction that involves the nitrogen of the amide group and the formation of non-volatile char [47].

The amount of ZnO nanostructures deposited on the veils was assessed by measuring the residual weight at the end of the heating cycle. The electrospun veils contained 3.65%, 29.82%, and 58.77% by weight of ZnO after a growth treatment of 1, 3, and 5 h, respectively. This evaluation, associated with the considerations previously reported during the SEM micrographs discussion, makes it possible to select optimal growth times. Additional information about the possible effect of the growth treatment on the electrospun fibers can be acquired by considering the derivative plot of TGA (Figure 5b).

The first derivative peak temperature (*T_max_*) for the neat electrospun veils was 431.80 °C [48] and a shift of this value was observed with the development of ZnO nanostructures. To establish the nature of this shift, the same analysis was carried out on specimens exposed to a pre-treatment with pure ethanol and on specimens subjected only to the seeding step. The pre-treatment determined a modification of the electrospun veil highlighted by a shift of *T_max_* at about 410.96 °C, while a much more significant shift (363.38 °C) was recorded after the seeding step. This effect can be ascribed to a combined action played by the absorption of water and ethanol that can act as plasticizers for nylon nanofibers [49], thus reducing their thermal stability [50]. While the attack by water can be directly at peptides that are hydrogen bridged, the alcohol molecules are large enough to weaken the intermolecular forces holding the polymer molecules together, leading to a further attack by the solvent and to a breakage of the intermolecular bridges between adjacent peptides.

In addition, the alkaline environment of the seeding step can cause hydrolysis of amide bonds [51,52]. The appearance of a new degradation step at the highest temperature (449.26 °C) after the seeding step, could be associated with the formation of electrospun fibers degradation by-products during the seeding/annealing treatment that showed a peak in the same region [53].

To confirm the chemical modification attributed to the alkaline conditions, the veil was treated by dipping it in ethanol and then in a sodium hydroxide solution at the same concentration adopted during the seeding step (0.16 mM) for 10 min followed by a drying step at 100 °C for other 10 min. The appearance of the peak at the highest temperature (~450 °C, Figure S3) confirmed the occurrence of the alkaline hydrolysis mechanism [54]. Nevertheless, the pretreatment used in this experimental study did not considerably affect the dimension of electrospun nanofibers that maintained an average diameter of 201.6 ± 71.1 nm and a rather smooth surface (Figure S4).

Considering the initial degradation temperature, a decrease from 402.74 °C to 290.20 °C after the pre-treatment, the seeding and 1 h of growth cycle, was observed. Additional growth cycles, which promoted the homogeneous coverage of the electrospun veils, resulted in an improvement in their thermal stability with a corresponding increase in the onset degradation temperature of 331.54 °C. This is because the presence of ZnO nanostructures acted as a protective layer that slows down the thermal decomposition of the decorated veils in accordance with other authors [17].

As one of the envisaged applications of these electrospun veils decorated with ZnO nanorods is as toughening elements in composite laminates, a preliminary investigation about the effect of this hydrothermal treatment on veils mechanical response was performed. Tensile testing was carried out to characterize the neat electrospun veils and decorated veils at selected growth times (1 h, 3 h, and 5 h), with the results summarized in Table 1.

A tensile test of the nanofiber veil allows to assess the average mechanical properties of the nanofibers rather than measuring an individual nanofiber and several factors are known to affect the resulting mechanical properties, including geometrical factors such as the number of crossings per nanofiber, the total nanofiber crossings in the veil and three-dimensional joints morphology, and the nanofiber molecular structure and orientation [55]. The addition of ZnO nanorods appreciably increased the tensile strength and, to a lower degree, the Young’s modulus of the as-received membranes up to 3 h growth time. As discussed in the morphology section, the homogeneous coverage of ZnO nanorods over the fibers’ surface can create linkages between them, leading to enhancement in stiffness of the membranes. The high fiber-to-fiber entanglements and resulting friction forces in the fibers assembly led to an increased resistance to slippage under loading. These effects can counteract, at least in a limited range of tensile strains, the side-effects caused by the hydrothermal treatment in terms of plasticization and depolymerization of the polymer nanofibers as highlighted by the thermogravimetric studies, which eventually resulted in an overall increase in elongation at break. Figure S5 shows the typical stress vs. strain curves of the electrospun veils, where an initial part exhibits a linear elasticity followed by nonlinear elasticity and a high resistance to deformation, especially for decorated veils, thus supporting cohesive forces among the nanofibers and enhanced resistance to slippage. A 5 h treatment was indeed characterized by a decrease in mechanical properties compared to the other treatment durations, but the final mechanical properties were not degraded compared to the untreated electrospun veils. This is likely due to much more significant effects of the treatment on the inherent structural properties of the polymer nanofibers, able to cancel out the benefits induced by the presence of ZnO nanorods.

In conclusion, the growth of ZnO structures on the surface of the electrospun veils was successfully developed and the growth time was identified as a crucial parameter: a duration lower than 3 h does not ensure a complete coverage of the veils, while a time longer than 5 h does not allow a precise control of ZnO growth and promoted the appearance of deposits on the nanofibers’ surface along with excessive degradation of the polymer structure.

#### 3.1.2. Method 2

**Figure 6:**
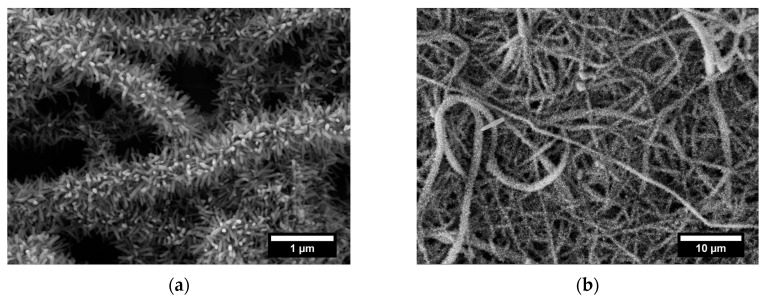

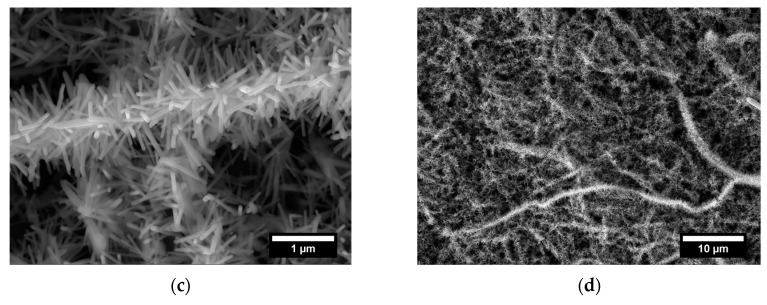
SEM micrographs at different magnification of electrospun veils decorated with ZnO after (**a**,**b**) 3 h and (**c**,**d**) 5 h of growth treatment in Method 2 with 75 mL as volume of growth solution.

**Figure 7:**
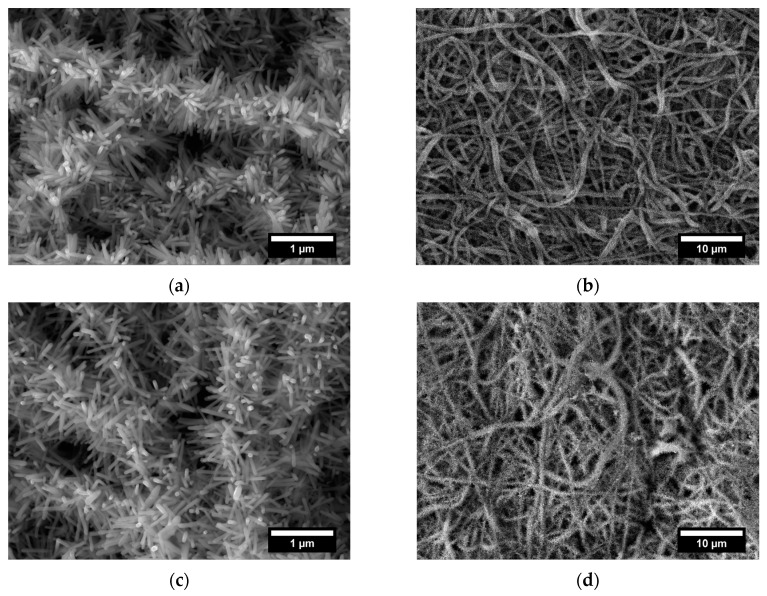
SEM micrographs at different magnification of electrospun veils decorated with ZnO after (**a**,**b**) 3 h and (**c**,**d**) 5 h of growth treatment in Method 2 with 250 mL as volume of growth solution.

The idea of developing a treatment suitable for commercial electrospun veils requires, at the same time, an easily scalable procedure. For this reason, a different growth technique (Method 2) was preliminary proposed and investigated. The specimens, after the pre-treatment and the seeding, were maintained in the same growth solution and the presence of ZnO on the surface was investigated by analyzing the SEM micrographs provided in Figure 6 and Figure 7. Based on the results previously reported for Method 1, two different growth times (3 h and 5 h) were chosen and the effect of two volumes of growth solution was studied.

The growth process was ensured and the ZnO nanorods maintained the hexagonal shape as shown in SEM micrographs at higher magnification (Figure 6a,c and Figure 7a,c). When a volume of 75 mL of solution was used, the diameter values ranged from 55.8 nm ± 11.6 nm after 3 h to 60.3 nm ± 13.7 nm after 5 h (Figure S6). Similar difference in diameter values was detected also when a volume of 250 mL was adopted: 53.5 nm ± 5.7 nm and 62.2 nm ± 12.5 nm were recorded after 3 h and 5 h of treatment, respectively (Figure S7). As for the length, after 3 h of treatment, values of 315.8 nm ± 54.8 nm and 375.4 nm ± 37.8 nm were achieved by exposing the veils to 75 mL and 250 mL of growth solution, respectively, showing a further increase on the axial dimension.

After 5 h of treatment a non-significant improvement in the length of ZnO nanorods was observed: an increase of 3.40% (75 mL) and a reduction of 8.04% (250 mL) compared to the specimens produced at 3 h were calculated. Therefore, an increase in the growth time did not lead to a significant increase in the height of the structures, thus defining 3 h as a sufficient time to maximize the growth of the nanorods.

A higher aspect ratio was exhibited by samples treated for 3 h in Method 2 condition compared to Method 1. The measured values were 5.13, 5.66, and 6.97 for ZnO nanorods grown for 3 h in Method 1 and in Method 2 with 75 and 250 mL, respectively.

Ultimately, by operating with Method 2 procedure a higher axial dimension than Method 1 at the same operative time (3 h) was guaranteed and the development of deposits was prevented, obtaining a more homogeneous specimens without clusters. This is in accordance with the discussion about the dependence of precipitation phenomena to temperature variations that in Method 2 are avoided since there is no manipulation during the synthesis.

**Figure 8:**
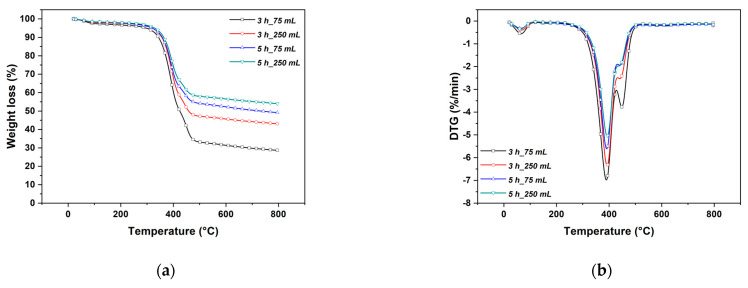
(**a**) Thermograms and (**b**) first derivative weight loss of neat electrospun veil and ZnO-decorated veils at different growth treatment times in Method 2.

The general thermal behavior was not altered in samples obtained with the Method 2, as can be inferred by comparing Figure 5 with Figure 8. However, a slight increase in the peak temperature was detected with a ZnO yield, after 3 h with 250 mL, equal to 43.03%, higher than the one obtained with the same duration in Method 1. The increase in thermal stability compared to Method 1 samples is ascribed to a lower degradation of the polymer structure, as confirmed by tensile test results. Numerical values are summarized in Table 1 while the typical stress vs. strain curves are reported in Figure S8. In fact, the mechanical properties of samples treated for 3 h with both volume solutions are higher than the corresponding values of samples in Method 1. Even in Method 2, a 5 h treatment, irrespective of volume solution, resulted in a decrease of tensile strength and Young’s modulus, which suggests limiting the treatment time to 3 h, which represents an optimal balance between mechanical properties and ZnO yield. The hexagonal wurtzite structure of ZnO was not affected by the method used, as similar XRD spectra were found also for the Method 2, where a slightly more developed crystalline structure can be observed for samples treated after 3 h in 250 mL (Figure 4b).

The high aspect ratio of ZnO nanorods after 3 h in 250 mL makes the electrospun veils suitable as a composite toughening element. In this regard, BET analysis showed that a higher surface area was obtained after the growth process of ZnO nanostructures. Indeed, values ranging from 8.99 m^2^/g to 12.00 m^2^/g for the neat and ZnO-decorated veils, respectively, after a 3h-growth process in 250 mL were recorded.

In conclusion, the success obtained in the decoration of commercial veils allows introducing possible future application regarding their use as toughening material in composites. Their use in a neat form proved to reduce the extension of delaminated area after low velocity impact test [32] while the presence of ZnO nanorods on fiber surface enhances the adhesion between fibers and polymer matrix [56,57].

### 3.2. Optical Properties

**Figure 9:**
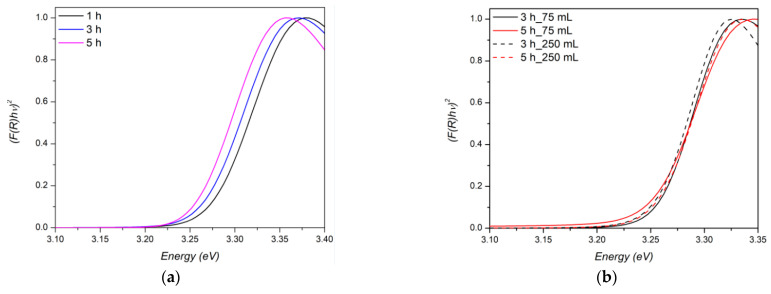
Direct-bandgap Tauc plots of veils decorated with ZnO with (**a**) Method 1 and (**b**) Method 2 procedures.

The ZnO nanorods grown on electrospun veils were studied by diffuse reflectance (UV-vis-DRS) spectroscopy. The optical gap value (*E_g_*) was estimated relying on the Kubelka–Munk method combined with the Tauc relation, as follows [58,59]:

[(*αhv*) = *B*(*hv* − *E_g_*)*^n^*], (7)

where *B* is a constant, *α* is absorption derived by the remission function of Kubelka–Munk *F*(*R*^∞^) (*α* = *k*(*λ*)/*s*(*λ*) = (1 − *R*^∞^)^2^/2*R*^∞^. Here, *R*^∞^ is defined as *R*^∞^ = *R*_sample_/*R*_reference_ with *R*_reference_, the diffuse reflectance measured for the Spectralon standard), *E_g_* is the average band gap of the material, *n* depends on the type of transition (*n* = 1/2 for direct transmission and *n* = 2 for indirect transmission), *h* is the Plank’s constant (6.626 × 10^−34^ J s), and *ν* is the frequency of photons. The direct average bandgap transition energies were estimated by extrapolating the straight-line segment to *α* = 0 of the (*αhν*)^2^ versus *hν* plot, as shown in Figure 9.

From the plots, it is observed that the transition between the edges of valence and conduction band is around 3.2 eV, which represents the optical energy band gap of the ZnO semiconductor [60]. When ZnO nanostructures were obtained by Method 1 procedure (Figure 9a), the *E_g_* decreases from 3.274 to 3.255 eV with increasing growth time compared to those synthesized by Method 2 (Figure 9b) where an almost constant *E_g_* value (3.25 eV) was obtained.

As previously reported [61,62], the *E_g_* value of the ZnO nanostructures systematically decreases due to an increase in the crystallite size and in the aspect ratio of the nanostructures [63]. Indeed, as previously assessed in the morphological discussion, the aspect ratio value ranged from 3.11 to 5.13 with the increasing of growth time following the Method 1 procedure. When Method 2 was adopted, this parameter was slightly influenced by the volume of growth solution: the measured value ranged from 5.66 to 6.97, further confirming the soundness of Method 2 as a growth procedure. In this regard, it has to be mentioned that also the presence of defects in the ZnO nanorods can generate energy levels between the valence band and conduction band, resulting in a lower *E_g_* value [64].

### 3.3. Photocatalytic Activity

Apart from the potential enhancement of interlaminar resistance in composite laminates, the presence of photoactive structures makes these materials attractive for environmental applications since they promote the production of radical species useful for the removal of pollutants. Furthermore, the presence of veil acts as a support for the oxides avoiding their release in the bulk liquid. The photosensitivity properties of electrospun veils decorated with ZnO were investigated by photocatalytic degradation tests of methylene blue (MB) as a model compound under UV irradiation. This test was possible because the hydrothermal synthesis adopted acted as modification of electrospun veils [28] that became hydrophilic with an almost null contact angle measured with distilled water.

**Figure 10:**
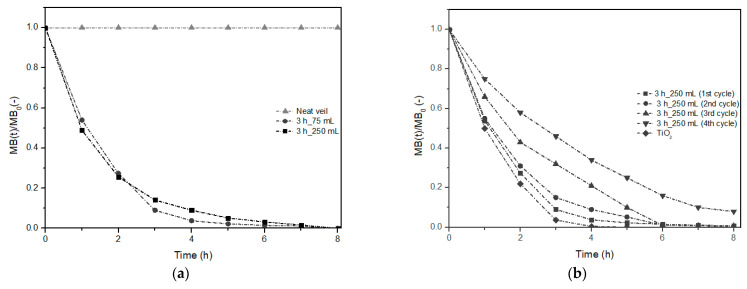
First kinetic-order methylene blue (MB) degradation curve under photocatalytic test with (**a**) a neat (triangles) and two different electrospun veils decorated with ZnO after 3 h of growth time (Method 2) and (**b**) after different use cycles and comparison with TiO_2_ (Degussa P25).

The effectiveness of the process was evaluated by monitoring the concentration of MB by the disappearance of the absorption peak at 666 nm. In Figure 10, the results of MB removal by the electrospun veils decorated with ZnO after 3 h of growth time (Method 2) at different volumes of growth solution are reported as mean values of three replications.

The good photocatalytic properties of both specimens tested can be observed in Figure 10a, where the degradation curves as MB(*t*)/MB_0_ are included. The MB removal of 86.0 ± 3.4% and 91.0 ± 5.1% for the samples treated in 75 mL and 250 mL, respectively, were measured already after 3 h of photocatalytic test. The complete degradation was reached after 7 h of exposure under UV light in both tests. TOC analysis confirmed the complete removal of the dye with a mineralization up to 100% at the end of the treatment.

These results were compared with those obtained in a blank test with a neat electrospun veil that revealed no adsorption and mineralization phenomena. This confirmed that the removal of MB in the presence of hexagonal ZnO nanorods can be attributed to the ability of such structures to promote the production of radical species such as superoxide anions (^•^O_2_^−^) and hydroxyl radicals (^•^OH) [65] thanks to the electron-hole pairs mechanism [24]. No substantial difference between the specimens was observed: in both cases a kinetic of first order (Equation (8)) well describes the MB removal time (*t*) in accordance with other authors [17,18,19,20,21,22,23,24,25,26,27,28,29,30,31,32,33,34,35,36,37,38,39,40,41,42,43,44,45,46,47,48,49,50,51,52,53,54,55,56,57,58,59,60,61,62,63,64,65,66], with a constant (*k*) of 0.65 h^−1^ ± 0.04 h^−1^ and 0.63 h^−1^ ± 0.06 h^−1^ for the veils 3_75 and 3_250, respectively.

MB(*t*)/MB_0_ = exp^(−*kt*)^, (8)

To highlight the goodness of the results obtained, a comparison with the results reported in literature by analogous studies has been performed considering the initial MB concentration adopted and the kinetic constant obtained by first order data fitting (mg/L min^−1^). Rahimi and Yazdani evaluated the photocatalytic activity of ZnO nanorods synthesized by different methods. In particular, their best result was observed by treating MB (8 mg/L) with 20 mg of ZnO powder for a total amount of 5 h with a degradation rate of 7.12 × 10^−2^ mg/L min^−1^ [59]. Fragalà and co-workers tested ZnO nanorods for photocatalytic MB (4.8 mg/L) removal and obtained a degradation rate of 2.1 × 10^−2^ mg/L min^−1^ [67]. In our tests the range of the degradation rate was 1.95 × 10^−1^ mg/L min^−1^ and 1.89 × 10^−1^ mg/L min^−1^ for the tests with veils 3 h_75 mL and 3 h_250 mL, respectively, indicating that the long time spent for the photocatalytic tests can be attributable to the large MB amount (18 mg/L) used in this study.

A difference in MB degradation rate was observed in the experiments conducted with the two tested samples. When the electrospun veil was treated with 250 mL of growth solution, the high availability of Zn complexes resulted in an increase of ZnO nanostructures (previously confirmed by SEM analysis) and, as a consequence, a larger production of radicals.

A further comparison was done by using a commercial nano-photocatalyst as Degussa P25 (Figure 10b). Under the same operating conditions and with an amount of catalyst equal to that used in the test with the ZnO nanorods, the MB degradation followed a similar trend to those obtained with both decorated veils, thus confirming the radical mechanism previously discussed and the good quality of the catalyst synthesized with the hydrothermal technique. The commercial catalyst (P25) made it possible to obtain a faster removal process than that obtained with the decorated veils highlighted by the kinetic constant value of about 1.32 h^−1^ ± 0.03 h^−1^.

In the attempt to use the veils for multiple treatment cycles, the removal efficiency of MB was measured after up to 4 cycles of reuse of the veils (Figure 10b). A decrease in MB degradation was revealed and after 3 h of photocatalytic test it was found equal to 85.0 ± 1.2%, 68.0 ± 3.5% and 54.0 ± 2.2% for a number of cycles of 2, 3, and 4, respectively. A significant decrease in first order kinetic process was observed during the third cycle use. Although during the second cycle a similar kinetic trend compared to the first use cycle was obtained (*k* = 0.59 h^−1^ ± 0.01 h^−1^), a decrease in kinetic constant value of 31.74% (0.43 h^−1^ ± 0.01 h^−1^) and 49.20% (0.32 h^−1^ ± 0.02 h^−1^) with respect to the first cycle was measured during the third and fourth cycle, respectively. In terms of degradation rate, 1.77 × 10^−1^ mg/L min^−1^, 1.29 × 10^−1^ mg/L min^−1^ and 9.60 × 10^−2^ mg/L min^−1^ were calculated for the second, third, and fourth cycle test, respectively, maintaining high values compared to those found in literature [59,60,61,62,63,64,65,66,67]. The comparison with commercial TiO_2_ that, in the adopted conditions, showed a degradation rate of about 3.93 × 10^−1^ mg/L min^−1^, suggests that, for this kind of applications, further optimization of the material is necessary to achieve good removal rate in short time and a stable removal efficiency after several applications.

## 4. Conclusions

The objective of this work was to evaluate the feasibility of decorating commercial electrospun veils with ZnO nanorods for two different purposes: the production of hierarchical materials to be used as toughening elements in composite laminates to prevent the occurrence of delamination and/or membranes suitable for photocatalytic water remediation applications. The growth of ZnO nanorods on electrospun veils was successfully performed by considering two different growth procedures: the contact of specimens with a growth solution refreshed every hour for a maximum time of 5 h (Method 1) and the treatment with different volumes of growth solution (75 mL and 250 mL) without refreshing the solution for a maximum time of 5 h (Method 2). A complete and homogeneous coverage of the specimens with well-aligned single crystalline wurtzite ZnO nanorods was obtained, with 70.5 nm ± 11.5 nm and 362.4 nm ± 104.6 nm as diameter and length of the nanorods after 3 h of treatment, respectively. In such condition, the precipitation phenomena were prevented and a final yield of 29.82% of ZnO was obtained. A detailed thermal and mechanical characterization highlighted that this optimized hydrothermal growth method was not detrimental to the mechanical properties of the veils even though a reduced thermal stability occurred due to hydrolysis of the polymer nanofibers. Considering the application as interleaves in composite laminates, the onset of thermal degradation was found to be higher than the typical curing temperatures of thermoset matrices. A possible development of this procedure was implemented by evaluating the effect of a different synthesis modality (Method 2) on ZnO growth. A good coverage degree of the electrospun veils was observed after 3 h of treatment by using 250 mL of growth solution without refreshing the solution. In such condition, homogeneous hexagonal structures with 53.5 nm ± 5.7 nm and 375.4 nm ± 37.8 nm as diameter and length were produced, respectively, by identifying Method 2 as a valid methodology to be adopted on a large scale. The higher aspect ratio obtained using Method 2 was linked to a high surface area that resulted, for this novel hierarchical nanocomposite, in good photocatalytic properties during the treatment of a solution of MB. After 7 h of treatment, complete mineralization of the pollutant was obtained. As regards the reusability of the decorated veils, up to the second cycle of use the MB removal followed the first kinetic order trend by maintaining a similar kinetic constant value. A substantial decrease in photocatalytic performance was observed during the fourth cycle where a reduction of 49.20% in kinetic constant value was calculated. Further optimization regarding veil dimension, light source and operative conditions are necessary to pursue this purpose.

## Figures and Tables

**Table 1:** Summary of mechanical properties of neat and decorated veils by Method 1 and Method 2 at different growth times.

	Growth Time (h)	Young’s Modulus (MPa)	Tensile Strength (MPa)	Elongation at Break (%)
Neat	-	100.6 ± 7.3	4.5 ± 0.3	15.5 ± 0.4
Method 1	1 h	102.4 ± 9.1	6.3 ± 0.6	19.7 ± 3.4
	2 h	102.6 ± 11.2	6.3 ± 0.5	18.0 ± 4.4
	5 h	76.7 ± 1.7	4.5 ± 0.3	15.9 ± 0.7
Method 2	3 h_75 mL	112.8 ± 11.6	8.0 ± 0.3	16.3 ± 1.2
	5 h_75 mL	115.9 ± 3.2	7.0 ± 0.9	12.1 ± 1.1
	3 h_250 mL	128.5 ± 3.1	8.5 ± 0.1	16.30 ± 0.17
	5 h_250 mL	126.50 ± 8.28	7.21 ± 0.12	10.4 ± 0.89

## Data Availability

The data presented in this study are available on request from the corresponding author.

## Supplementary Materials

The following are available online at https://www.mdpi.com/2079-4991/11/2/418/s1, Figure S1. SEM micrograph and corresponding EDX analysis of ZnO seed deposited nylon nanofibers. Figure S2. Normal distribution of diameter and length of ZnO nanostructures in Method 1 at different growth treatment times. In detail, diameter distribution after (a) 1 h, (b) 3 h and (c) 5 h growth treatment times and length distribution after (d) 3 h and (e) 5 h growth treatment times. Figure S3. Thermogram (black curve) and first derivative weight loss (red curve) of electrospun veil after a treatment in ethanol and in a sodium hydroxide solution at the same concentration adopted during the seeding step (0.16 mM). Figure S4. (a,b) SEM micrographs at different magnifications of commercial electrospun veil after a sequential pretreatment with ethanol and NaOH (0.16 mM) and (c) corresponding diameter distribution. Figure S5. Typical tensile stress vs. strain curves of as-received and ZnO-decorated electrospun veils in Method 1. Figure S6. Normal distribution of diameter and height of ZnO nanostructures at (a,b) 3 h and (c,d) growth treatment times in Mode 2 with 75 mL as volume of growth solution. Figure S7. Normal distribution of diameter and height of ZnO nanostructures at (a,b) 3 h and (c,d) growth treatment times in Mode 2 with 250 mL as volume of growth solution. Figure S8. Typical tensile stress vs. strain curves of ZnO-decorated electrospun veils in Method 2.
