# Supplementary material for: Functionalization of Commercial Electrospun Veils with Zinc Oxide Nanostructures
Source: Nanomaterials (Basel). 2021 Feb 6;11(2):418. doi: 10.3390/nano11020418 (PMC7916010; doi:10.3390/nano11020418)
Supplement: Supplementary file 1 [file nanomaterials-11-00418-s001.pdf]

nanomaterials

Supplementary Materials

Functionalization of Commercial Electrospun Veils with Zinc Oxide Nanostructures

Irene Bavasso [1,*], Francesca Sbardella [1], Maria Paola Bracciale [1], Matteo Lilli [1], Jacopo Tirillò [1], Luca Di Palma [1,*], Anna Candida Felici [2] and Fabrizio Sarasini [1]

[1] Department of Chemical Engineering Materials Environment, Sapienza-Università di Roma & UdR INSTM, Via Eudossiana 18, 00184 Roma, Italy; francesca.sbardella@uniroma1.it (F.S.); mariapaola.bracciale@uniroma1.it (M.P.B.); matteo.lilli@uniroma1.it (M.L.); jacopo.tirillo@uniroma1.it (J.T.); fabrizio.sarasini@uniroma1.it (F.S.)

[2] Department of Basic and Applied Sciences for Engineering, Sapienza-Università di Roma, Via Scarpa 16, 00161 Roma, Italy; annac.felici@uniroma1.it

* Correspondence: irene.bavasso@uniroma1.it (I.B.); luca.dipalma@uniroma1.it (L.D.P.)

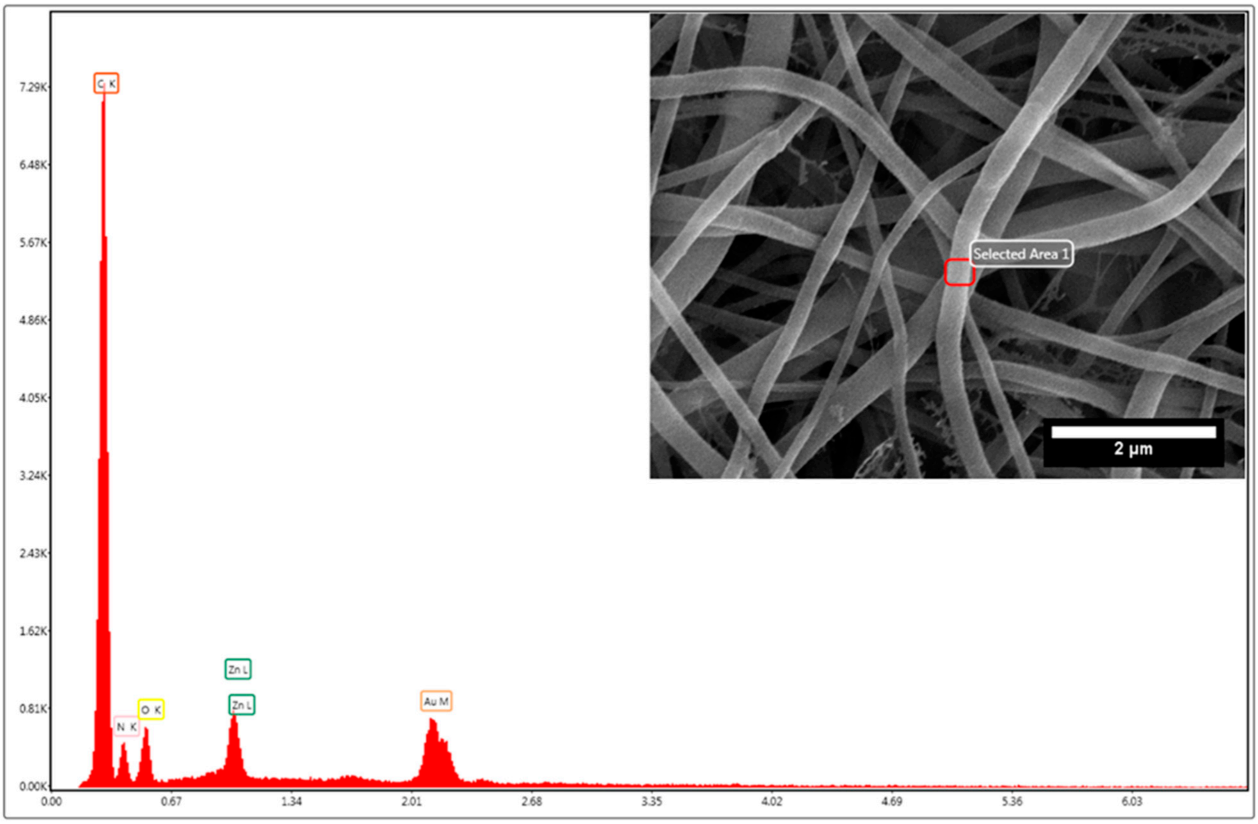

Figure S1. SEM micrograph and corresponding EDX analysis of ZnO seed deposited nylon nanofibers.

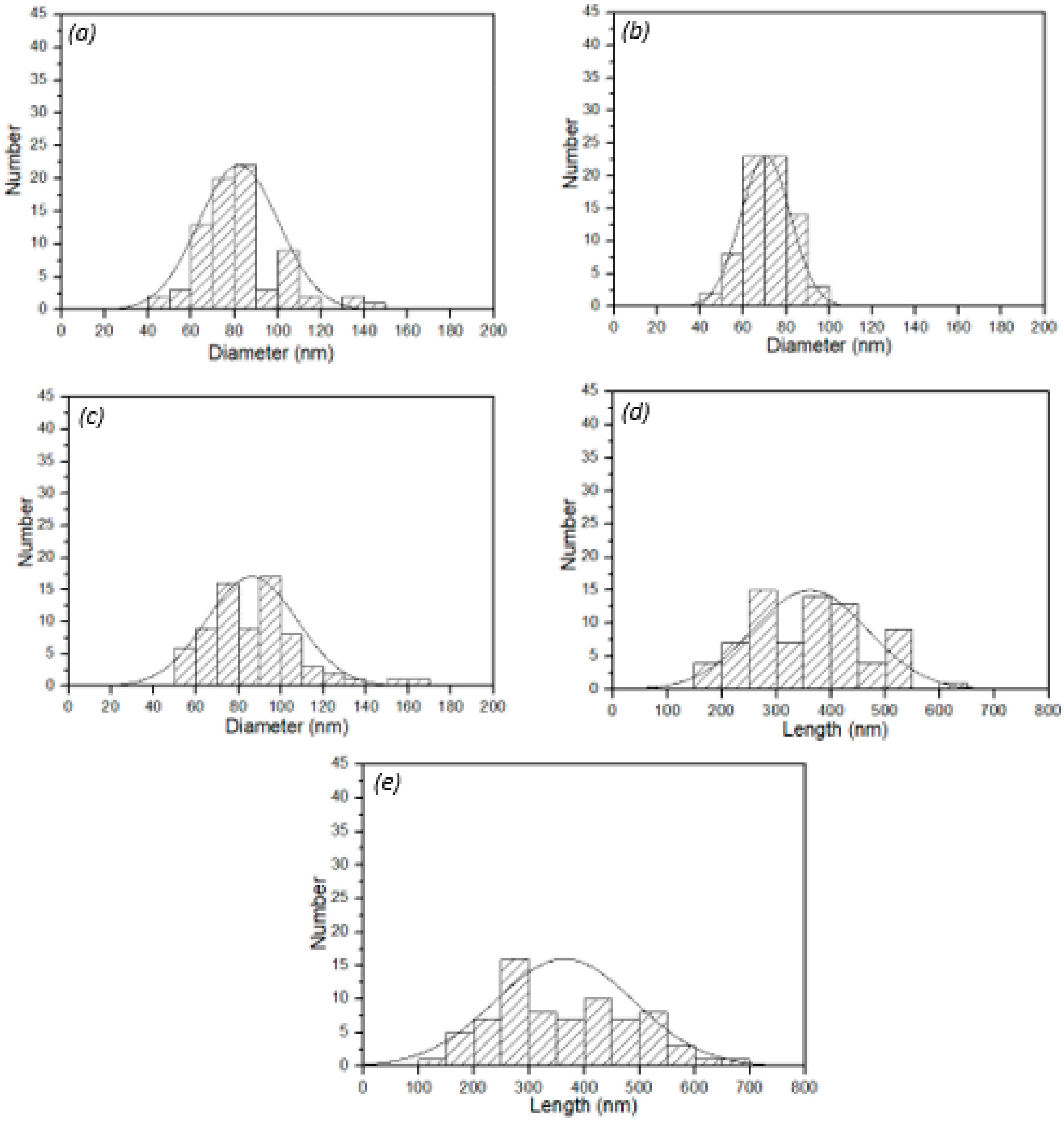

Figure S2. Normal distribution of diameter and length of ZnO nanostructures in Method 1 at different growth treatment times. In detail, diameter distribution after (**a**) 1 h, (**b**) 3 h and (**c**) 5 h growth treatment times and length distribution after (**d**) 3 h and (**e**) 5 h growth treatment times.

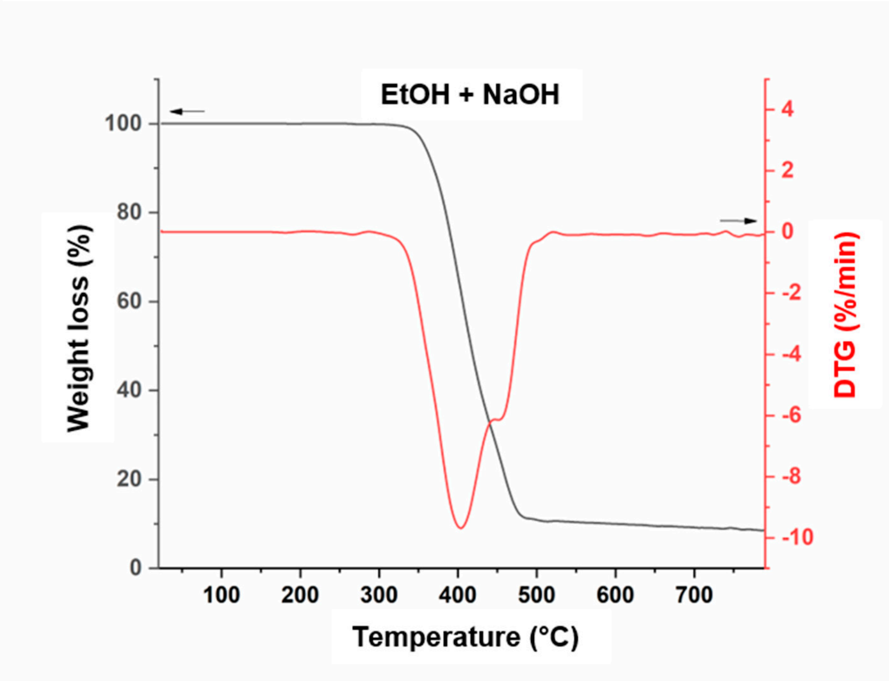

Figure S3. Thermogram (black curve) and first derivative weight loss (red curve) of electrospun veil after a treatment in ethanol and in a sodium hydroxide solution at the same concentration adopted during the seeding step (0.16 mM).

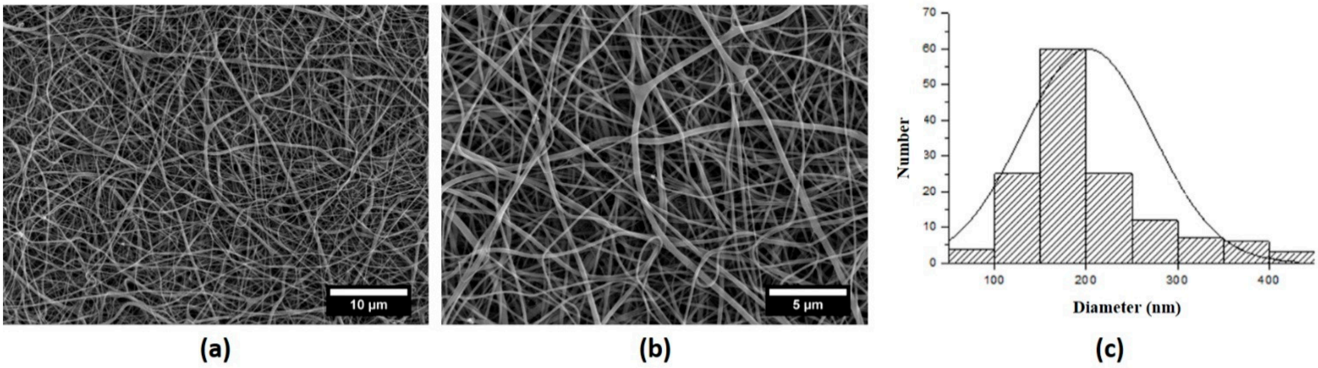

Figure S4. (**a**,**b**) SEM micrographs at different magnifications of commercial electrospun veil after a sequential pretreatment with ethanol and NaOH (0.16 mM) and (**c**) corresponding diameter distribution.

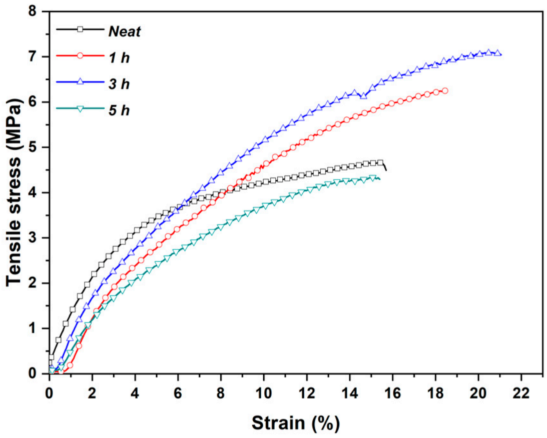

Figure S5. Typical tensile stress vs. strain curves of as-received and ZnO-decorated electrospun veils in Mode 1.

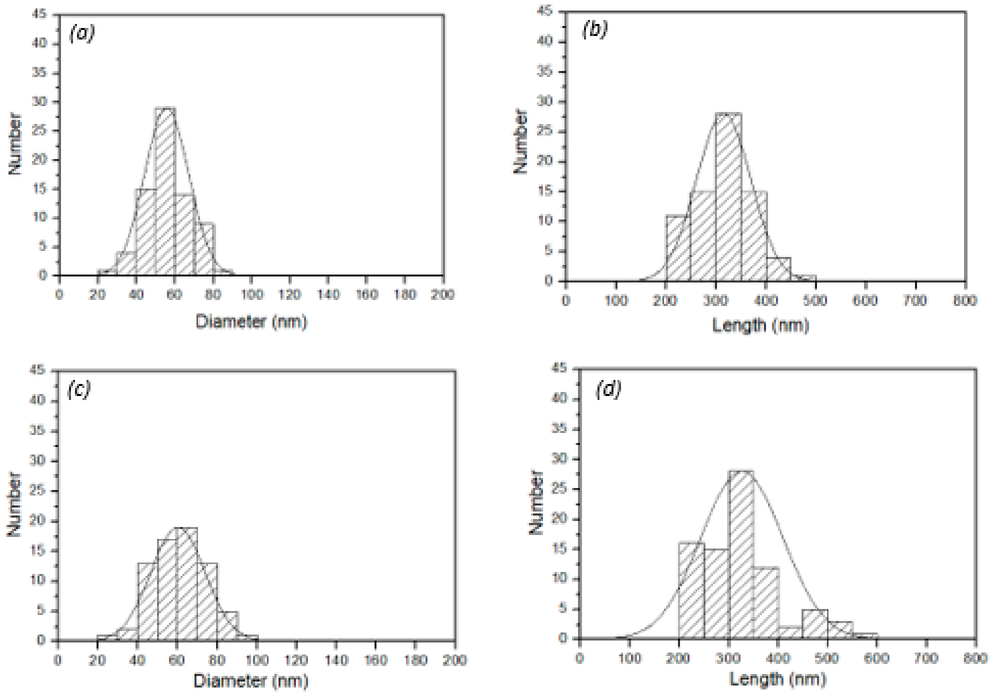

Figure S6. Normal distribution of diameter and height of ZnO nanostructures at (**a**,**b**) 3 h and (**c**,**d**) growth treatment times in Mode 2 with 75 mL as volume of growth solution.

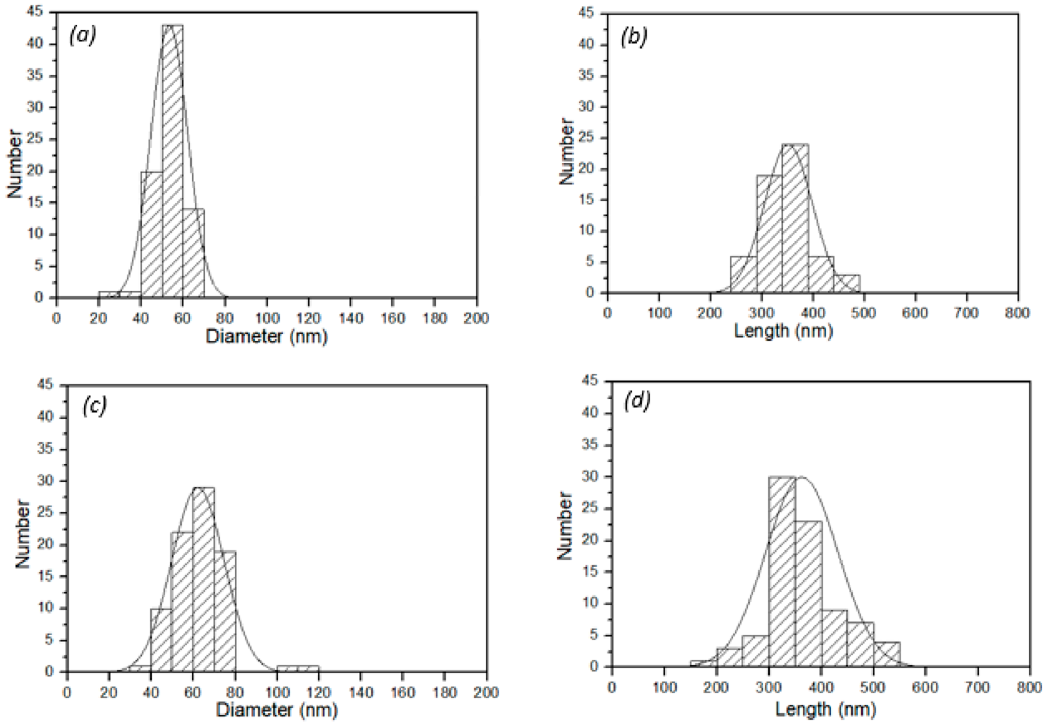

Figure S7. Normal distribution of diameter and height of ZnO nanostructures at (**a**,**b**) 3 h and (**c**,**d**) growth treatment times in Mode 2 with 250 mL as volume of growth solution.

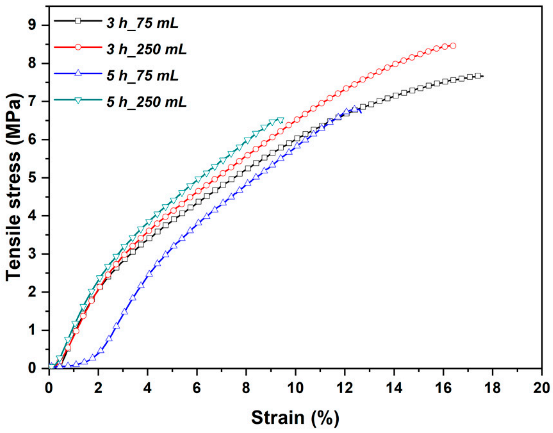

Figure S8. Typical tensile stress vs. strain curves of ZnO-decorated electrospun veils in Mode 2.
